# Acute Renal Failure Secondary to Inadvertent Propylene Glycol Overdose with Single-Day High-Dose Vitamin D (Stosstherapy)

**DOI:** 10.1155/2019/1482727

**Published:** 2019-01-20

**Authors:** David H. Jelley, Deborah Zayneb Mohamad Ali, Michelle Condren

**Affiliations:** ^1^Pediatric Department, University of Oklahoma School of Community Medicine, Tulsa, 4444 E 41^st^ St., Tulsa, OK 74135, 918-619-4803, USA; ^2^Pediatric Department, University of Oklahoma School of Community Medicine, Tulsa, 4502 E 41^st^ St, Tulsa, OK 74135, 918-660-3400, USA; ^3^University of Oklahoma College of Pharmacy, Pediatric Department, University of Oklahoma School of Community Medicine, Tulsa, 4502 E. 41^st^ St, Tulsa, OK 74135, 918-660-3578, USA

## Abstract

Globally, there has been increasing attention paid to vitamin D deficiency and its treatment. Vitamin D stosstherapy with high-dose ergocalciferol in a single day is reemerging as a potential treatment option. We present an as yet unreported complication of acute renal injury due to propylene glycol toxicity in a 7-month infant treated with vitamin D stosstherapy. The product label for the vitamin D used for this patient states it is dissolved in propylene glycol (PG), but the amount is not listed on this or other US ergocalciferol liquid products that contain PG. Caution should be used when considering vitamin D stosstherapy with liquid ergocalciferol products available in the United States.

## 1. Introduction

Vitamin D is essential for calcium homeostasis and bone health and is obtained from sun exposure, vitamin supplements, and foods fortified with vitamin D including dairy products, infant formula, and breakfast cereals. Once foods were fortified, nutritional or vitamin D deficiency was thought to be a disease of the past, yet it remains common. While mild vitamin D deficiency is generally asymptomatic in children, more severe deficiency can result in rickets with or without hypocalcemia. Treatment includes vitamin D supplementation plus adequate nutritional intake of calcium. Stosstherapy, or single-day high dose vitamin D therapy, has been used since the 1930s and may be warranted when patients present with acute conditions secondary to low vitamin D requiring more rapid resolution of deficiency, or when there are concerns about compliance with daily therapy. Stosstherapy has been reported to be a safe and effective method of treatment [1]; however, concerns have been raised regarding the markedly elevated vitamin D levels which occur with this form of treatment. Furthermore, unintended consequences related to other molecules in the drug formulation may occur; in this case propylene glycol results in acute renal failure following vitamin D stosstherapy in an infant with nutritional rickets.

## 2. Case

**Figure 1:**
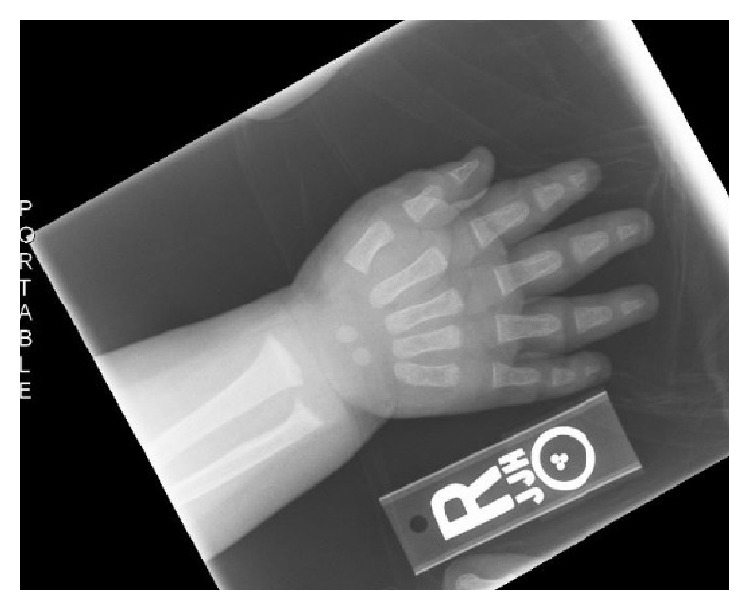
Classic rachitic changes of the wrist: fraying and widening at the radial and ulnar metaphyses.

A previously healthy 7-month-old African-American male presented to the ER in late winter after a generalized tonic-clonic seizure. There was no history of trauma or febrile illness. He was exclusively breastfed but had only received a multivitamin supplement for the first three months of life. Physical exam revealed a postictal infant with short stature, length = 64 cm (<5th percentile) and weight of 9.2 kg (75th percentile). Neurologic exam demonstrated brisk patellar reflexes, and the remainder of his physical exam was unremarkable. Initial labs showed severe hypocalcemia with total serum calcium = 5.9 mg/dl (normal range 8.5-10.9 mg/dl) and ionized calcium = .67 mmol/L (normal range 1.18-1.29 mmol/L). He was treated with IV calcium, after which serum calcium levels rose slightly but remained significantly below the normal range. Subsequent evaluation showed an undetectable 25-hydroxy vitamin D level of less than 4 ng/ml, with elevated parathyroid hormone and alkaline phosphatase levels (Table 1). Skeletal survey showed rachitic changes (Figure 1).

The patient was diagnosed with nutritional rickets and was administered vitamin D stosstherapy treatment with ergocalciferol (Virtus Pharmaceuticals, ergocalciferol oral solution, USP, 8,000 international units per mL) 100,000 international units given orally every 2 hours for a total of 600,000 international units over 12 hours. By the last dose, the patient had become lethargic and urine output was decreased from 2.1 cc/kg/hr before treatment to .7 cc/kg/hr after treatment. Labs revealed acute renal failure, metabolic acidosis, and hyperkalemia. Creatinine level rose from .41 mg/dl pretreatment to 3.0 mg/dl following stosstherapy over a 36-hour timespan. He was treated with fluid resuscitation, diuretics, sodium polystyrene sulfonate, bicarbonate, and continued IV calcium. Renal function and calcium levels normalized, and serum creatinine returned to baseline of .41 mg/dl and total calcium = 9.8 mg/dl. The patient was discharged home after 4 days on oral vitamin D and calcium supplementation.

## 3. Discussion

To our knowledge, this is the first reported case of renal failure due to vitamin D stosstherapy. Derived from the German word stossen, meaning “to push”, stosstherapy consists of high-dose (600,000 units) ergocalciferol given over a single day. Use of this therapy for rickets was first reported over 75 years ago and is now regaining popularity [1–4]. For decades all infants in the German Democratic Republic (GDR) received 600,000 units ergocalciferol every 3 months for the first 18 months of life. While a significant percentage of these infants had elevated vitamin D levels and hypercalcemia, none were reported to have suffered renal injury or nephrocalcinosis [3, 5]. While renal failure has been associated with chronic vitamin D toxicity when dosed on a daily or weekly basis, we found no reports associated with single day stosstherapy [6].

Post hoc analysis of the ergocalciferol preparation carried by our hospital found that the side panel states it is dissolved in propylene glycol (PG), but the amount is not listed on this or other US ergocalciferol liquid products that contain PG. Further investigation revealed that the above product contains 103.6 g/100 mL of propylene glycol. The infant received 600,000 units of vitamin D2, equivalent to 75 mL of solution or 77.7 g of propylene glycol. According to the World Health Organization, this far exceeds the maximum tolerable amount of 25 mg/kg/day (or 227.5 mg for the baby in this event). In fact, the baby was exposed to 340 times the maximum amount [7]. Propylene glycol is a clear, colorless, odorless, and tasteless product used as a stabilizer, thickener, and texturizer. When given in large doses, drugs containing PG induce metabolic acidosis and reversible acute renal failure [8–11]. The primary difference between this case and previous reports using high doses of vitamin D is use of a concentrated form of ergocalciferol which did not contain propylene glycol; therefore, we suspect that acute renal failure in our patient was secondary to propylene glycol (PG) toxicity.

Caution should be used when using vitamin D stosstherapy to avoid liquid formulations of ergocalciferol solubilized with propylene glycol.

## Figures and Tables

**Table 1:** Serial laboratory values from admission through six-week followup appointment.

	**Admission**	**Post Stosstherapy**	**Discharge**	**Outpatient followup**	**Normal Values**
	**Feb 11**	**Feb 14**	**Feb 18**	**March 28**	
**Calcium**	5.9	7.0	9.8	10.2	8.0-10.5 mg/dL
**Ionized calcium**	0.67	0.88	1.18		1.18-1.29 mmol/L
**Phosphorus**	5.3	5.6	6.0		3.8-6.2 mg/dL
**Magnesium**	2.1	2.2	1.6		1.7-2.9 mg/dL
**Vitamin D**	<4	112	350	86	30-100 ng/ml
**Alkaline phosphatase**	876	905	746	307	150-420 U/L
**PTH**	404.8	582.3	40	40	7.2-111.9 pg/mL
**Creatinine**	0.41	3.00	0.41	.45	0.20-0.73 mg/dL
**CO**_**2**_	17	14	26	21	20-24 mmol/L
